# A mutation in the *FOXE3* gene causes congenital primary aphakia in an autosomal recessive consanguineous Pakistani family

**Published:** 2010-03-30

**Authors:** Iram Anjum, Hans Eiberg, Shahid Mahmood Baig, Niels Tommerup, Lars Hansen

**Affiliations:** 1 Human Molecular Genetics Laboratory, Health Biotechnology Division, National Institute for Biotechnology & Genetic Engineering (NIBGE), Faisalabad, Pakistan; 2 The Wilhelm Johannsen Centre for Functional Genome Research, ICMM, Panum Institute, University of Copenhagen, Blegdamsvej 3b, DK-2200 Copenhagen N, Denmark; 3 Section IV, Department of Cellular and Molecular Medicine, The Panum Institute, University of Copenhagen, Blegdamsvej 3b, DK-2200 Copenhagen N, Denmark

## Abstract

**Purpose:**

Aphakia is the complete absence of any lens in the eye, either due to surgical removal of the lens as a result of a perforating wound or ulcer, or due to a congenital anomaly. The purpose of this study was to elucidate the molecular genetics for a large consanguineous Pakistani family with a clear aphakia phenotype.

**Methods:**

The initial homozygosity screening of the family was extended to all the known autosomal recessive cataract loci in order to exclude the possibility of surgical cataract removal leading to aphakia. The screening was performed using polymorphic nucleotide repeat markers, followed by DNA sequencing of a possible candidate gene, the forkhead box protein E3 gene (*FOXE3*). The identified mutation was counter-checked by a diagnostic restriction enzyme digest of all the family members, and an analysis of the normal population.

**Results:**

The initial homozygosity screening of 13 known autosomal recessive loci resulted in negative LOD (logarithm of odds) scores. The aphakia phenotype suggested a mutation in *FOXE3* close to the AR-locus 1p34.3-p32.2, and sequence analyses revealed the nonsense mutation c.720C>A, changing cysteine 240 to a stop codon. Segregation in the family was shown by diagnostic restriction enzyme digest, and marker analysis of another aphakia family from Madagascar carrying the same mutation excluded the presence of a founder mutation. Clinical re-examination of the family was not possible due to the escalating security concerns and internal displacement of the population in this region of Pakistan which has prevailed for many months.

**Conclusions:**

*FOXE3* is responsible for the early developmental arrest of the lens placode, and the complete loss of a functional *FOXE3* protein results in primary aphakia. It can also be deduced that this mutation is quite primitive in origin since the same mutation is responsible for the same phenotypic outcome in two families of geographically different descent.

## Introduction

Primary congenital aphakia is a rare congenital disorder that has been classified histologically into primary and secondary forms. Primary aphakia appears due to the early developmental arrest of the lens placode leading to the complete absence of the lens, while secondary aphakia is observed in cases where the lens is formed initially but is subsequently resorbed perinatally (OMIM 610256). The phenotypic outcome is quite diverse in these two forms because of the different stages of onset. In primary aphakia, the missing lens formation leads to severe congenital eye malformations, including aplasia of the interior eye segment, whereas secondary aphakia leads to less severe ocular defects.

Primary congenital aphakia is known to be caused by mutations in the forkhead box protein E3 (*FOXE3*) gene in both humans and mice [1-6]. *FOXE3* is a member of the forkhead family of genes; more than 40 *FOX* genes are known in the human genome and they are transcription factors characterized by an 80–100 amino acid DNA binding forkhead motif. The human *FOXE3* maps to chromosome 1p33, and was initially named *FREAC-8* (forkhead-related activator 8) or *FKHL12* (forkhead, drosophila, homolog-like 12) [7]. The Fox proteins exhibit very high functional diversity, and are involved in very early key developmental processes, including the formation of the notochord and the establishment of the body axis, carnio-pharyngeal development, hair development, hearing, and speech and language [8], and several Fox proteins have been shown to be expressed during eye development [9]. The function of *FOXE3* in lens development has been extensively studied in mice where homozygous null mutations result in congenital aphakia with the absence of lens development [1,2,10,11]. In humans, homozygous *FOXE3* mutations have been associated both with recessive inherited congenital primary aphakia [3,4], and the dominant inheritance of ocular dysgenesis, cataracts and Peters’ anomaly [5,6]. Here, we report the characterization of a *FOXE3* mutation identified in a consanguineous Pakistani family that results in primary congenital aphakia.

## Methods

### Biologic sample

A large inbred family with congenital primary aphakia was ascertained from a remote village of Pakistan (Basti Moza Kotla Mosa, District Bahawalpur, South Punjab) having many affected individuals. The mode of inheritance as evident from the segregation of disease alleles in the pedigree was autosomal recessive. Venous blood samples were collected from fifteen members of the family, depending on their availability and willingness to participate in the study. Genomic DNA was extracted following the standard phenol:chloroform method.

### STS marker analysis

All known autosomal recessive cataract loci were enlisted (Table 1) and initially screened using two or more STS (Sequence-Tagged-Site) marker systems for each locus in order to exclude the possibility of cataract involvement in the phenotype. A 3-primer STS marker protocol was developed for the fragment analyses using ABI3130*XL* and GeneMapper 3.0 technology (Applied Biosystems, Foster City, CA). The 3-primer labeling system uses a FAM labeled primer (FAM-TGA CCG GCA GCA AAA TT), and the identical primer sequence was added 5′ to one of the genome specific PCR primers. All oligonucleotides were purchased from TAG Copenhagen (Copenhagen, Denmark). Briefly, for the 3-primer protocol, the primer concentrations were: FAM-primer 0.8 μM, forward extended primer 0.1 μM, and reverse primer 0.25 μM applying standard PCR conditions using Ampliqon III Taq polymerase (Ampliqon, Copenhagen, Denmark). The PCR conditions were as follows: pre-denaturation 95 °C, 10 min; then 30 cycles 95 °C, 1 min; 60 °C (or specific annealing temperature tested by temperature gradient), 1 min; 72 °C, 1 min followed by 8 cycles 95 °C, 1 min; 50 °C, 1 min; and 72 °C, 1 min. Total PCR volumes were adjusted to 12 μl using 10–20 ng template DNA. Two point LOD scores were calculated using the LIPED program [12].

**Table 1:** List of autosomal recessive cataract locus and STS markers.

**Locus position**	**Markers selected**	**Gene**	**Ethnicity**	**Reference**
1p34.3-p32.2	D1S255, D1S2892, D1S197	Unknown	Pakistan	[13]
1q21.1	D1S442, D1SGJA5-GJA8*	*GJA8* (*CX50*)	South India	[14]
3p22–24.2	D3S1298	Unknown	Arab	[15]
6p24.2	D6S470, D6S1034	*GCNT2*	Arab	[16]
9q13-q22	D9S768, D9S152	*CAAR*	Pakistan	[17]
14q24.3	D14S986, D14S1025, D14S1047, D14S273	*CHX10*	Turkey, UAE	[18]
16q22.1	D16S3019, D16S3086, D16S421, D16S3107, D16S3095	*HSF4*	Pakistan, China, Tunisia	[17,19]
19q13	D19S416	Unknown	Pakistan	[20]
19q13.33	D19S246	*LIM2*	Iraqi Jewish	[21]
20p12.1	D20S860	*BFSP1*	India	[22]
22q11.23	D22S421	*CRYBB3*		[23]
22q12.1	D22S315, D22S1167	*CRYBB1*	Israel	[24]
21q22.3	D21S1411, D21S1890, D21S1885	*CRYAA*	Jewish Persian	[25]

### DNA sequencing of *FOXE3*

This gene consists of a single exon and was sequenced for genomic variants by PCR amplification using overlapping sets of primers (Table 2) covering the coding region. This was followed by direct DNA sequencing on ABI3130 XL using BigDye ver1.1 technology (Applied Biosystems). The sequences were checked for possible variants using ChromasPro (Technelysium Pty Ltd., Tewantin, Australia) by alignment to the reference sequences (GenBank NM_012186 and NP_036318). The sequencing primers were purchased from TAG Copenhagen, and the Taq DNA polymerases from Qiagen (Hilden, Germany) and Invitrogen (Carlsbad, CA). The PCR products were analyzed by 2% agarose gel-electrophoresis, 1× TBE and the DNA was stained with ethidium bromide.

**Table 2:** PCR sequencing primers for *FOXE3*.

**Forward primer**	**Reverse primer**	**PCR length**
FOXE3_ex1.1f TGTCCATATAAAGCGGGTCG	FOXE3_ex1.1r ATGTACGAGTAGGGCGGCTT	298 bp
FOXE3_ex1.2f TTCTCTGGCTTCCCTGCC	FOXE3_ex1.2r TCGGTGATGAAGCGGTAGAT	272 bp
FOXE3_ex1.3f AAGCCGCCCTACTCGTACAT	FOXE3_ex1.3r TCGTTGAGCGTGAGATTGTG	170 bp
FOXE3_ex1.4f TTCATCACCGAACGCTTTGC	FOXE3_ex1.4r AGGAAGCTGCCGTTGTCGAA	185 bp
FOXE3_ex1.5f AAGGGCAACTACTGGACGCT	FOXE3_ex1.5r TAGCTCCGGCTGCAGGTT	267 bp
FOXE3_ex1.6f TCTGTTCAGCGTCGACAG	FOXE3_ex1.6r CAGGTCGCACAGGTGCC	351 bp

### Restriction enzyme digest

The mutation was counter-confirmed using a restriction enzyme digest with DdeI (New England Biolabs, Ipswich, MA) of the PCR product generated by the primer pair FOXE3–1.6 (Table 2) under standard conditions in a volume of 20 µl, and the cleaved products were analyzed by 2% agarose gel-electrophoresis, 1× TBE and the DNA was stained with ethidium bromide.

## Results and Discussion

**Figure 1:**
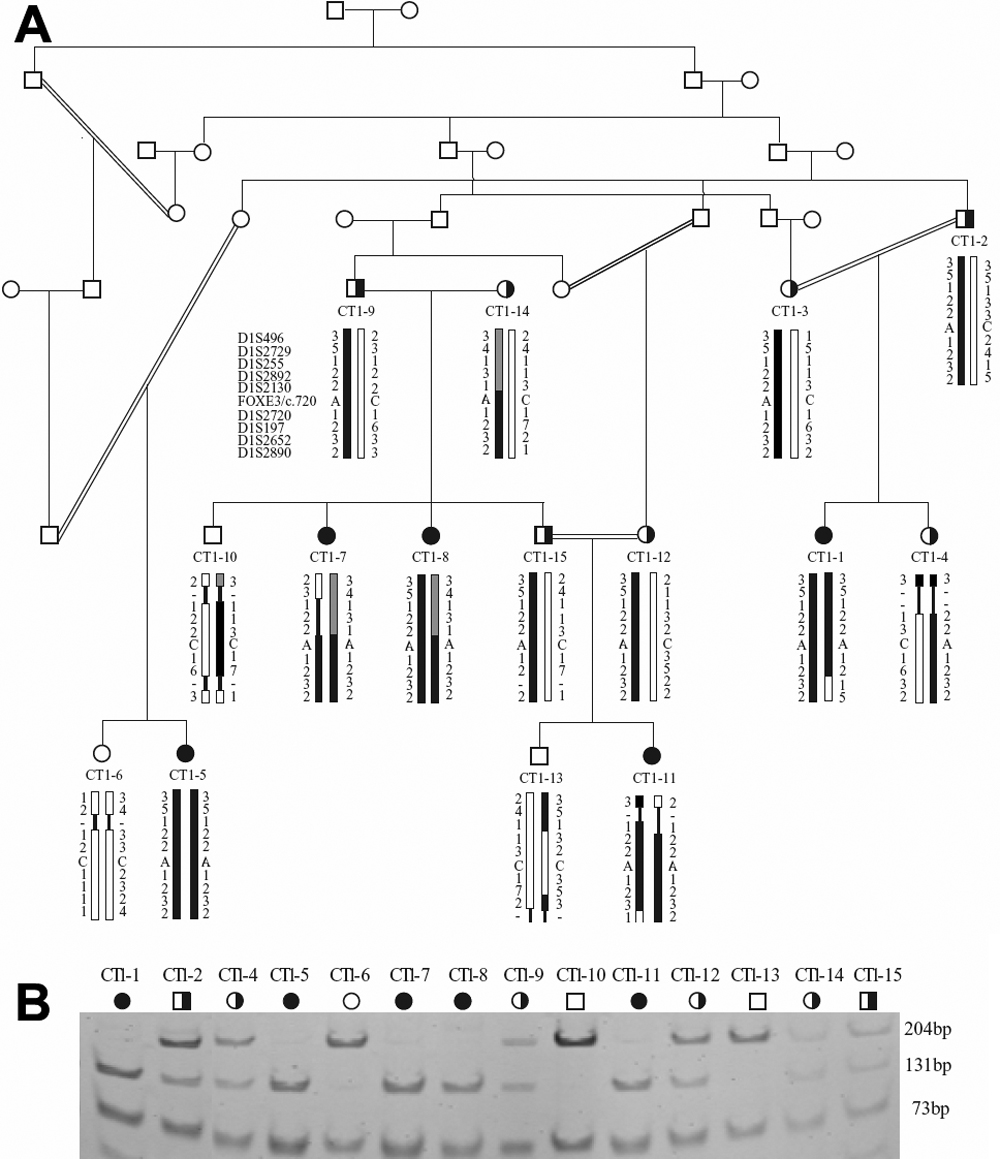
Pedigree and haplotype analysis of CT1. **A**: The pedigree of family CT1 shows a high degree of consanguinity in 7 generations. Two haplotypes are represented in the pedigree; the spouse CT1–14 carries the identical mutation and shares the identical haplotype distal to the *FOXE3* gene. The two haplotypes are shown as black or black and gray. Symbols: open circles or squares are healthy individuals, filled symbols represent affected individuals and half filled symbols represent carriers. **B**: Restriction enzyme digests of all the family members where DNA was available demonstrate the segregation of the mutation with the disease in the family. The restriction enzyme DdeI recognizes the mutation and cleaves the 204 bp band into two fragments of 131 bp and 73 bp. An additional 75 bp band is observed in all individuals.

All the available DNA samples for family CT1 were genotyped for all possible autosomal recessive cataract loci (Table 1) in order to rule out the possibility of cataract involvement in the resulting aphakic eyes. Initial homozygosity was traced on chromosome 1p33 by STS markers D1S255, D1S2892, and D1S197. Haplotype analysis based on more adjacent markers revealed several polymorphisms throughout the family which helped to identify a narrow conserved region around the *FOXE3* gene (Table 3 and Figure 1A). All the affected individuals presented homozygous alleles except for individuals CT1–7 and CT1–8, whereas the phenotypically normal individuals were either carriers of heterozygous alleles or homozygous. Interestingly, part of the disease haplotype was even brought in from outside the main kindred by CT1–14, suggesting that either the carrier was related to the CT1 family, or that disease carriers are highly prevalent in the region. Individual CT1–14 carried the identical disease haplotype proximal to the *FOXE3* locus but a different haplotype distal to the *FOXE3* locus (Figure 1A), which suggested a recombination between the *FOXE3* locus and the marker D1S2130 (see Table 3). LOD score calculations both for the *FOXE3* mutation and the two STS markers (Table 4) demonstrated positive LOD scores, with a maximum of Z=6.62 at θ=0.0 for the *FOXE3* mutation.

**Table 3:** STS markers used for fine mapping of the homozygous region at 1p33.

**STS marker**	**Physical position chromosome 1 (hg18)**
D1S496	35,179,917
D1S2729	36,843,493
D1S255	37,422,301
D1S2892	39,963,503
D1S2130	41,590,073
FOXE3	47,654,331 - 47,656,311
D1S2720	47,680,375
D1S197	50,523,064
D1S2652	55,239,419
D1S2890	57,645,988

**Table 4:** LOD score for *FOXE3* mutations and markers.

**STS marker**	**θ**						
Order	0.0	0.01	0.05	0.1	0.2	0.3	0.4
D1S2130	2.40	2.30	2.00	1.70	1.00	0.50	0.10
c.720C>A	6.62	6.50	6.00	5.35	3.97	2.51	1.10
D1S2720	1.60	1.60	1.40	1.10	0.70	0.40	0.10

Mut p=0.001

Sequencing of the coding region of *FOXE3* in one affected individual using overlapping primer pairs (Table 2) revealed a C>A single base substitution (c.720C>A) leading to a nonsense mutation in the cysteine 240 codon (p.Cys240X) as the underlying genetic cause of the disease phenotype. The restriction enzyme DdeI (recognition site 5′-CTNAG-3′) was chosen to confirm the mutation which cleaves the wild type allele of 204 bp into fragments of 73 bp and 131 bp, respectively. Restriction enzyme cleavage of the family demonstrated segregation of the mutation with the disease trait, and carriers were heterozygote for the wild type and the mutant alleles (Figure 1B) confirming the recessive mode of inheritance adopted by mutation.

**Figure 2:**
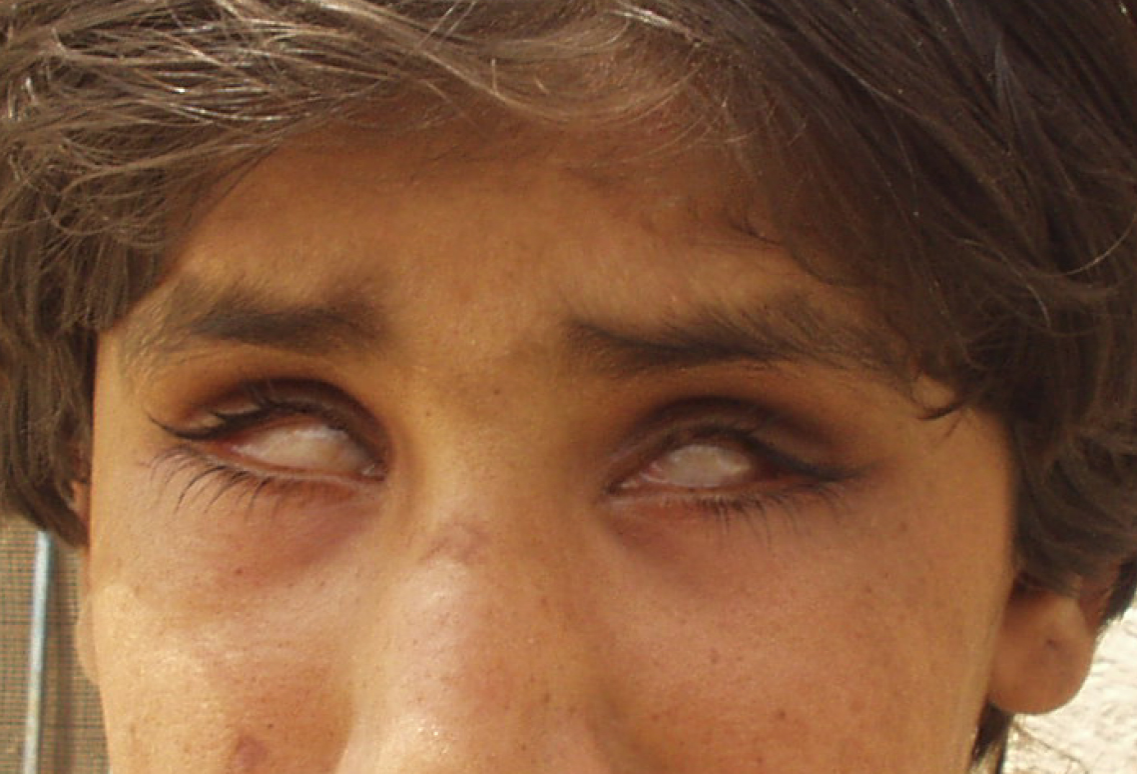
The individual CT1–11 showing complete congenital primary aphakia.

**Figure 3:**
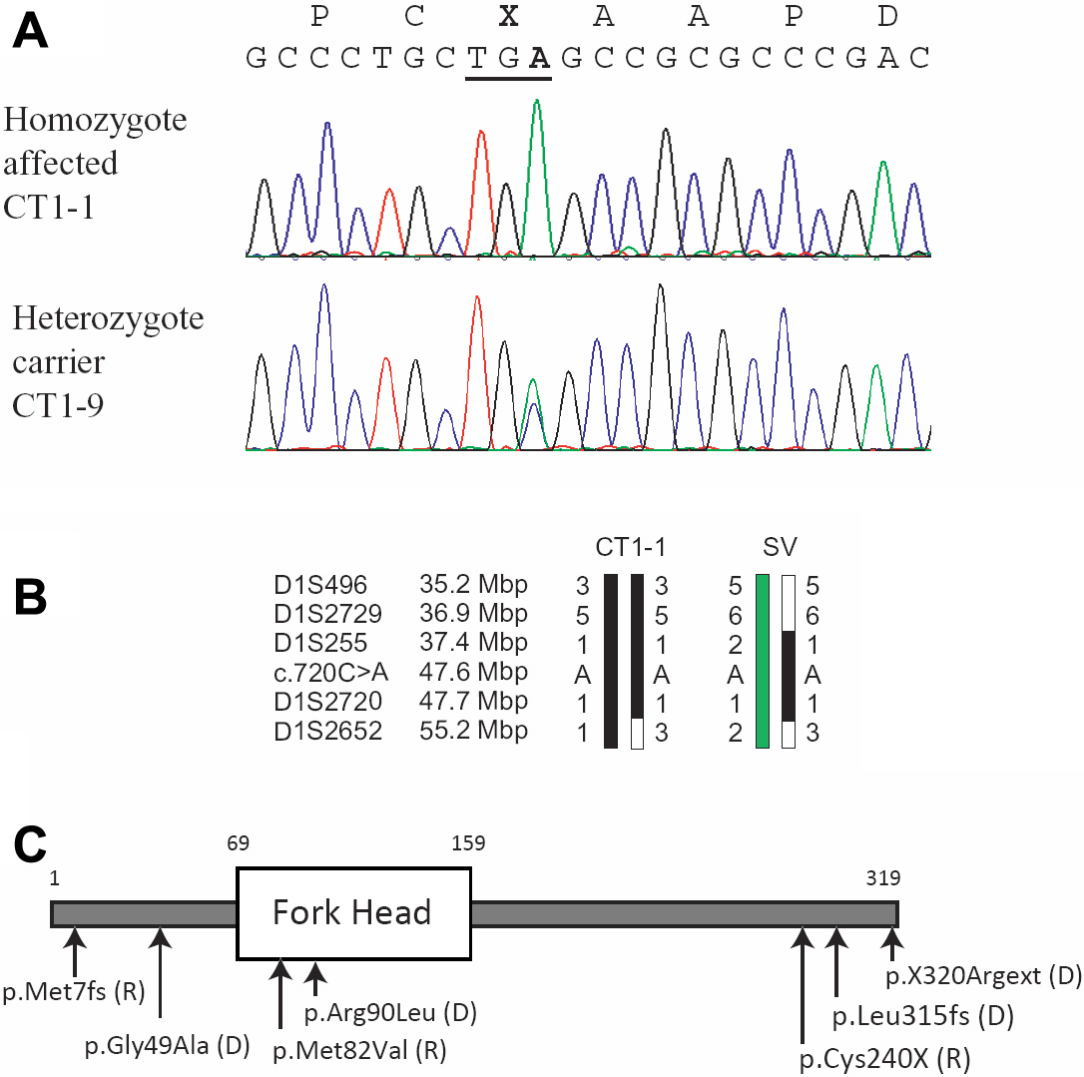
Genotype analysis of the *FOXE3* mutation. **A**: DNA sequencing chromatograms showing homozygote mutant and carrier status for two individuals. **B**: Microsatellite analysis for one affected form of the Pakistani and Madagascan families demonstrate segregation of different haplotypes in the two families which exclude an ancestral origin for the mutation p.Cys240X. **C**: Schematic presentation of the FOXE3 protein. Recessive inherited mutations (R) and dominant mutations (D) are clearly denoted [3-6].

The identical mutation was first reported in an inbred family from Madagascar [3]. Presenting the same underlying mutation, both families share the same phenotype showing the complete absence of the lens (Figure 2). Marker analyses were set for one individual from each of the families to see if the shared *FOXE3* mutation originated from the same ancestral founder. No informative SNPs were found in the nearby vicinity of *FOXE3* locus, and so several STS markers in the region were analyzed in one affected person from each family. The haplotype analysis demonstrated different haplotypes segregating in the two families (Figure 3B). As a consequence, it is very likely that the p.C240X mutation occurred independently in the two families.

The expression of the *FOXE3* gene is limited to the lens, but mutations in *FOXE3* result in various ocular phenotypes leading either to dominant or recessive inheritance [3-6]. Mutations in *FOXE3* have been reported in 8 families so far, including CT1 and a total of 7 different mutations have been identified (Figure 3C). Four mutations characterized in families with dominant inheritance are reported in combination with Peters’ anomaly, cataract and other ocular dysgenesis [4-6], and four families, including CT1, manifest recessive inheritance in association with the more severe phenotype primary aphakia [3,4].

All carriers in family CT1 were healthy as reported previously for the three other recessive families [3,4]. This suggests the pathogenic nature to be a null mutation with loss of function rather than haploinsufficiency as suggested earlier [4,5]. Unfortunately, it has not been possible to re-examine the CT1 family after identification of the mutation as a result of escalating security concerns, and internal displacement of the population in the region of Pakistan where the family resides.

*FOXE3* is a single exon gene encoding a 319 amino acid protein, and the recessive p.Cys240X mutation results in premature termination of translation and a truncated protein carrying the forkhead domain (Figure 3C). The very initial expression of *FOXE3* is observed in the lens-forming surface ectoderm (E 9.5), and maintains its presence throughout lens placode formation, and in later processes too as invagination and separation from the ectoderm above. Later during the development, the expression of *FOXE3* is switched off from the differentiating lens fiber cells, restricting itself to the anterior lens epithelium (E 14.5) where its expression remains confined throughout the life of the subject [2,4]. The complete lack of a functional FOXE3 protein product may explain the complete lack of lens development resulting in aphakia observed both in association with the p.Cys240X mutation found in two families as well as for the two other recessive mutations (Figure 3C). Finally, it is noteworthy that all the reported recessive families were consanguineous, and that three out of four were of Pakistani descent.
